# Interplay of soil characteristics and arbuscular mycorrhizal fungi diversity in alpine wetland restoration and carbon stabilization

**DOI:** 10.3389/fmicb.2024.1376418

**Published:** 2024-04-10

**Authors:** Hao Tang, Qian Li, Qian Bao, Biao Tang, Kun Li, Yang Ding, Xiaojuan Luo, Qiushu Zeng, Size Liu, Xiangyang Shu, Weijia Liu, Lei Du

**Affiliations:** ^1^Key Laboratory of Land Resources Evaluation and Monitoring in Southwest (Sichuan Normal University), Ministry of Education, Chengdu, China; ^2^The Faculty of Geography Resource Sciences, Sichuan Normal University, Chengdu, China; ^3^College of Resources, Sichuan Agricultural University, Chengdu, China; ^4^Sichuan Provincial Cultivated Land Quality and Fertilizer Workstation, Chengdu, China; ^5^Sichuan Academy of Forestry, Chengdu, China; ^6^State Key Laboratory of Biogeology and Environmental Geology, China University of Geosciences, Wuhan, China; ^7^Research Center for Carbon Sequestration and Ecological Restoration, Tianfu Yongxing Laboratory, Chengdu, China; ^8^Chengdu Academy of Agriculture and Forestry Sciences, Chengdu, China

**Keywords:** ecological restoration, carbon stabilization, arbuscular mycorrhizal fungi, tea bag index, climate change mitigation

## Abstract

Alpine wetlands are critical ecosystems for global carbon (C) cycling and climate change mitigation. Ecological restoration projects for alpine grazing wetlands are urgently needed, especially due to their critical role as carbon (C) sinks. However, the fate of the C pool in alpine wetlands after restoration from grazing remains unclear. In this study, soil samples from both grazed and restored wetlands in Zoige (near Hongyuan County, Sichuan Province, China) were collected to analyze soil organic carbon (SOC) fractions, arbuscular mycorrhizal fungi (AMF), soil properties, and plant biomass. Moreover, the Tea Bag Index (TBI) was applied to assess the initial decomposition rate (*k*) and stabilization factor (*S*), providing a novel perspective on SOC dynamics. The results of this research revealed that the mineral-associated organic carbon (MAOC) was 1.40 times higher in restored sites compared to grazed sites, although no significant difference in particulate organic carbon (POC) was detected between the two site types. Furthermore, the increased MAOC after restoration exhibited a significant positive correlation with various parameters including *S*, C and N content, aboveground biomass, WSOC, AMF diversity, and NH_4_^+^. This indicates that restoration significantly increases plant primary production, litter turnover, soil characteristics, and AMF diversity, thereby enhancing the C stabilization capacity of alpine wetland soils.

## 1 Introduction

Alpine wetlands offer critical ecosystem services, such as biodiversity conservation, water filtration, and flood control (Chen et al., 2013, 2022; Yang et al., 2017). Recently, alpine wetlands have been recognized for their ability to store large amounts of carbon (C) in soils, is crucial for global climate regulation and maintaining ecological balance (Chen et al., 2022). However, alpine wetland ecosystems face challenges from human disturbances, such as land-use change, drainage, and ditching, particularly grazing, which undermines the structural integrity and functionality, thereby impairing C storage capability (Lu et al., 2015; Guo et al., 2018; Sun et al., 2018).

The alpine wetlands situated on the Qinghai-Tibetan Plateau, represent one of the largest alpine wetland ecosystems, offering a striking illustration of the profound influence of livestock grazing on these fragile ecosystems (Sun et al., 2022; Wang et al., 2023; Zhang et al., 2023). The practice of grazing is deeply integrated into the local socio-economic practices, brings about significant alterations to various ecological aspects, including soil properties, rates of litter decomposition, and soil microbes (Sun et al., 2018; Ji et al., 2020; Qin et al., 2021; Wang et al., 2022; Liu Y. et al., 2023). These changes have cascading effects on the C cycle, for instance, grazing-induced disturbances can disrupt soil structure and nutrient dynamics, impacting both the quantity and quality of organic matter decomposition and subsequent soil C stabilization (Sun et al., 2018; Ji et al., 2020). Furthermore, grazing modifies the diversity and functionality of soil microbial communities, which are crucial for organic matter breakdown and C cycling, thereby influencing soil respiration rates and the overall C storage capacity of the soil (Qin et al., 2021; Liu Y. et al., 2023).

In response to these challenges, ecological restoration emerges as a pivotal strategy for conserving alpine wetlands in the climate change (Erwin, 2009; Zivec et al., 2023). Most restoration efforts focus on halting grazing to revive natural vegetation and environmental conditions, aiming to restore the original balance of wetland ecosystems. This approach is hypothesized to significantly boost the capability of C sequestration in wetland soils. Expected ecological benefits include the revival of native plant species, increased above-ground biomass, and enhanced litter input, vital for SOC stabilization (Law et al., 2017; Gao et al., 2021). However, the complex mechanisms through which ecological restoration influences C stabilization are multifaceted and not yet fully elucidated. Particularly, the roles of diverse microbial communities in these rehabilitated environments are critical yet underexplored aspects.

Restoration activities have the potential to alter soil microbial diversity and functionality, which are key drivers in organic matter decomposition and nutrient cycling – processes integral to the C sequestration capacity of wetlands (Zivec et al., 2023). Changes in microbial community structure can lead to variations in microbial processes such as litter decomposition, nitrification, and methanogenesis, each of which plays a significant role in the wetland C cycle (Yarwood, 2018; Li et al., 2023). Furthermore, restoration may influence the interaction between plants and soil microbes, including symbiotic relationships such as those involving arbuscular mycorrhizal fungi (AMF), which are crucial for nutrient uptake and organic matter transformation. The role of AMF becomes especially critical in this context, as they enhance soil health by improving plant access to essential nutrients, thereby facilitating more robust plant growth and a higher potential for carbon storage in these ecosystems (Moon et al., 2016). This underscores the importance of incorporating AMF dynamics into restoration strategies, aiming to maximize carbon sequestration in alpine wetlands and contribute effectively to global climate change mitigation efforts.

Therefore, field experiments were conducted in the Zoige alpine wetlands, a typical alpine wetland ecosystem in southwest China, to examine the ecological transition from grazing to restoration. By selecting representative sites for both grazing and restoration, our study aims to provide insights into ecosystem dynamics during this transition. For assessing the litter turnover, we used the Tea Bag Index (TBI) method, based on commercial green tea and rooibos tea, to measure the initial decomposition rate (*k*) and the stabilization factor (*S*) (Keuskamp et al., 2013; Tang et al., 2020). This innovative approach offers a standardized, efficient, and accurate means of measuring decomposition and carbon stabilization, distinguishing itself from traditional methods through its simplicity and cost-effectiveness. We also analyzed the soil properties, the SOC fractions, such as particulate organic carbon (POC) and mineral-associated organic carbon (MAOC), and the diversity of AMF. Our objective is to understand how C stabilization and AMF diversity differ under livestock grazing and restoration. We hypothesized that: (1) Restoration positively improves plant biomass, soil properties, litter turnover, and AMF diversity. (2) Restoration efforts will significantly increase both POC and MAOC formation due to the positive feedback of plant biomass, soil properties, stabilized of litter, and AMF diversity.

## 2 Methods

### 2.1 Site description and experimental design

**Figure 1:**
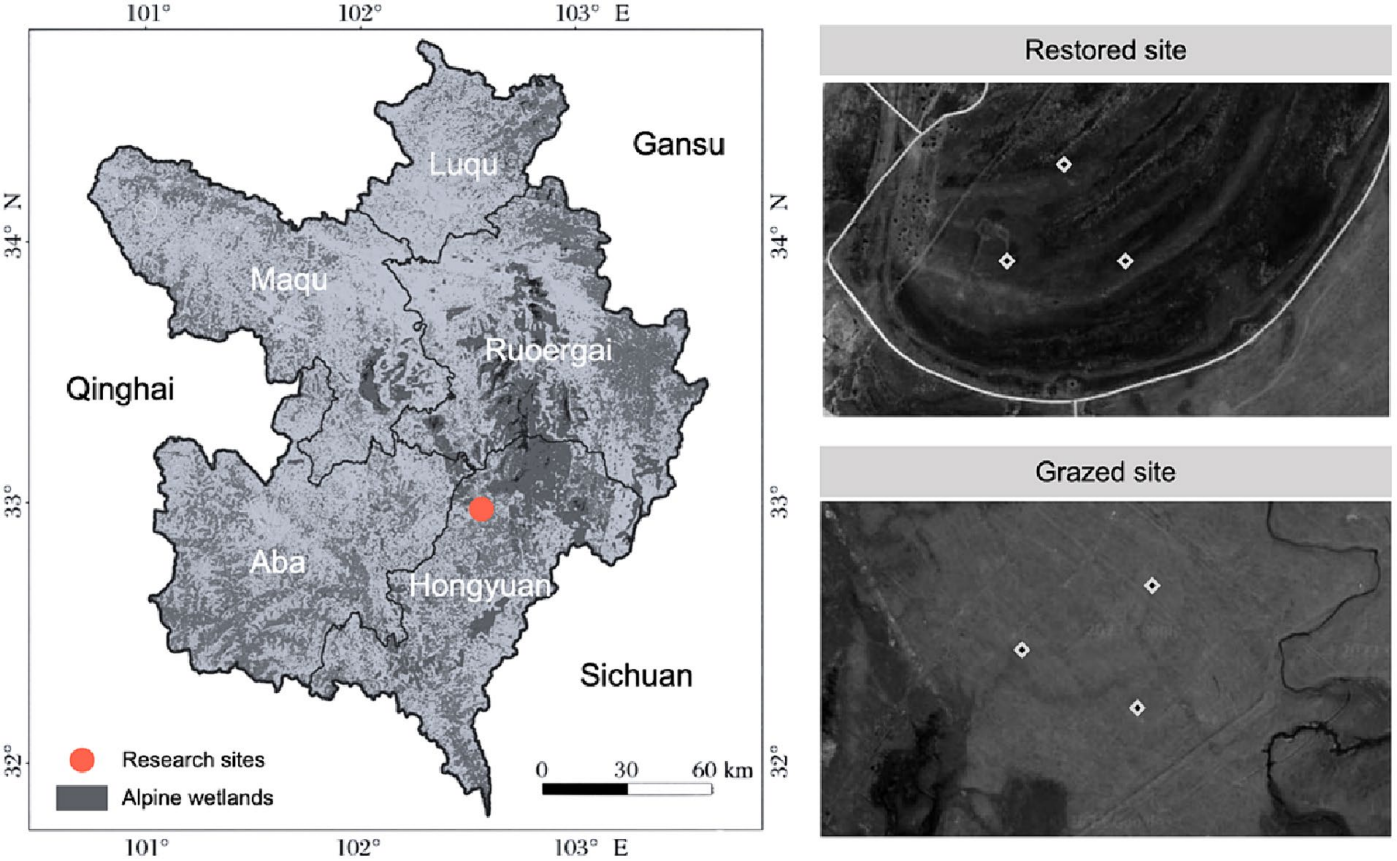
Location of experimental site and sub-plot distribution in grazed and restored plots. This figure illustrates the precise locations of the study areas, distinguishing between zones subjected to long-term grazing and those under ecological restoration for 2 years.

This research was conducted in the Zoige Alpine Wetland, located at 32.8096°N, 102.5568°E, near Hongyuan County in Sichuan Province, China (Figure 1). Situated on the eastern Tibetan Plateau, this extensive and diverse ecosystem is noted for its high elevation, typically between 3,400 and 3,600 meters. The wetland is predominantly covered by rich peatlands and marshes, featuring a variety of flora such as *Blysmus sinocompressus*, *Elymus nutans*, and *Kobresia pygmaea*. The region experiences a continental plateau cold temperate monsoon climate, characterized by long, cold, and dry winters, without a distinct summer season. The average annual temperature is around 1.4°C, with recorded extremes ranging from a high of 25.4°C to a low of −33°C. Annually, the area enjoys 2506.7 h of sunshine, and receives precipitation varying from 542 to 801 mm, mostly between May and October.

To investigate the impacts of grazing and ecological restoration on alpine wetland ecosystems, two adjacent areas with distinct land-use histories were selected. The first area, subjected to long-term grazing, and the second, under ecological restoration for 2 years, were chosen to serve as comparative study sites. In each area, three replicate experimental plots, each measuring 2 m x 2 m, were established based on criteria ensuring uniformity in soil type and vegetation cover. The selection criteria included historical land use, vegetation density, soil composition, and hydrological conditions to accurately represent the contrasting land-use scenarios. A total of six plots were utilized for the study. The precise locations and layouts of these plots are detailed in Figure 1, illustrating the methodical approach to selecting grazed versus restored sites and providing a clear basis for evaluating the restoration’s effects on these ecosystems.

### 2.2 Sampling collection and processing

To assess the potential differences in litter input and microbial substrate supply between grazed and restored sites, aboveground biomass was sampled in September 2023. This specific timing was chosen to coincide with the peak vegetative growth period in the study area, ensuring that the biomass measurements accurately reflected the maximum biological productivity for both site types within the annual growth cycle. Sampling during this period allows for a more precise comparison of the ecological impacts of grazing versus restoration on plant biomass accumulation.

In each plot, biomass samples were collected from a 40 cm x 40 cm quadrat, ensuring consistent sampling across all sites. These samples included all types of aboveground vegetation. After collection, the biomass samples were thoroughly washed, dried at 70°C for 72 h, and then weighed to determine the dry mass. Soil samples were extracted using a 15 cm diameter PVC corer, consistently to a depth of 20 cm at each site. From each core, a 20 g subsample was immediately preserved at −20°C for subsequent microbial diversity analysis. The rest of the soil was sieved using a 2.5 mm mesh and air-dried to constant weight. This process facilitated the determination of the dry mass and other key soil properties from the sieved material.

### 2.3 Analysis of soil properties

The measurement of specific soil parameters—ammonium (NH_4_^+^), nitrate (NO_3_^−^), water-soluble organic carbon (WSOC), soil moisture, electrical conductivity (EC), and pH—was meticulously conducted to provide a comprehensive evaluation of soil characteristics and nutrient dynamics, crucial for understanding ecological processes in grazed and restored sites. The concentrations of NH_4_^+^ and NO_3_^−^ in the soil were quantified using a colorimetric method (Saha et al., 2018). Soil extracts were prepared by mixing soil samples with 2 M KCl solution, followed by filtration. The extracts were then analyzed using a flow injection analyzer, which provided accurate measurements of NH_4_^+^ and NO_3_^−^ concentrations. WSOC was determined by extracting the soil with deionized water at a soil to water ratio of 1:5 (w/v). The mixture was shaken for 1 h and then centrifuged. The supernatant was filtered through a 0.45 μm filter, and the WSOC content was measured using a TOC analyzer (Tao and Lin, 2000). TC and TN content was determined by the element analyzer (Elementar-Analysensysteme GmBH, Germany). Soil moisture was assessed by weighing the soil samples before and after oven-drying at 105°C for 24 h. Soil EC and pH were measured in a soil-water suspension (1,5 soil to water ratio). The suspension was stirred and allowed to settle, after which the EC was measured using an EC meter and pH using a pH meter.

### 2.4 SOC fractionations

POC and MAOC in soil samples used a size-fractionation approach (Liu et al., 2022). Initially, soil samples were air-dried, gently crushed, and sieved to 2 mm to standardize the sample size. For POC isolation, a 6 g subsample was wet-sieved (5 g/L sodium hexametaphosphate solutions, 30 mL) with a 53 μm mesh, separating larger organic particles, which were then dried and weighed. The finer material passing through the sieve, representing the MAOC fraction, was similarly dried and weighed. Both fractions were analyzed for C content using a CNS analyzer (Elementar-Analysensysteme GmBH, Germany), which measures CO_2_ produced by combusting the samples.

### 2.5 TBI parameters

The TBI protocol, as described by Keuskamp et al. (2013), was employed to provide a standardized measure of decomposition. Commercial green tea (EAN: 8714100770542; Lipton, Unilever) and rooibos tea (EAN: 8722700188438; Lipton, Unilever) were utilized, chosen for the well-documented decomposition characteristics reported by Tang et al. (2021, 2023). Green tea, known for its relatively high nitrogen content and rapid decomposition rate, contrasts with rooibos tea, which decomposes more slowly due to its lower nitrogen content and higher lignin composition. The TBI method assesses two key parameters: *k*, indicating the decomposition speed of the labile fraction, and the stabilization factor *S*, which quantifies the proportion of the labile fraction that remains undecomposed and stabilized in the soil. The initial weight of the tea bags was determined by subtracting the average weight of five empty bags (green tea: 1.592 ± 0.004 g; rooibos tea: 1.801 ± 0.006 g) from the weight of the filled bags. These tea bags were buried in each site for 3 months at -15 cm soil depth (start in June, and end in September), after which they were retrieved, thoroughly cleaned, and dried for 48 h at 75°C to determine the final weight. The *k* and *S* values of the tea material were calculated according to the methodology outlined by Mueller et al. (2018), involving W_r_(t), the post-incubation weight of the rooibos tea (t in days), a_r_ for the labile fraction of the rooibos tea, and 1-a_r_ for its recalcitrant fraction. a_g_ represents the labile fraction of the green tea, while H_g_ and H_r_, as reported by Tang et al. (2023), represent the labile fractions of the green and rooibos teas, respectively.

$$W_{r}\left(t\right)=a_{r}e^{-kt}+\left(1-a_{r}\right)$$

$$S=1-a_{g}/H_{g}$$

$$a_{r}=H_{r}\left(1-S\right)$$

### 2.6 Soil DNA extraction and lllumina sequencing of soil AMF community

Soil DNA was extracted from a 500 mg sample using the FastPure Soil DNA Isolation Kit (Bio-pharm Technology Co., Ltd., Shanghai, China). The AMF 16S rRNA gene was amplified by PCR using specific primers AMV4-5NF (AAGCTCGTAGTTGAATTTCG) and AMDGR (CCCAACTATCCCTATTAATCAT) as described by Van Geel et al. (2014), utilizing a GeneAmp 9700 thermocycler (ABI, Thermo Fisher, Waltham, MA). PCR reactions were performed in triplicates in a 20 μL mix, including 4 μL of 5 × FastPfu Buffer, 2 μL of 2.5 mM dNTPs, 0.8 μL of each primer (5 μM), 0.4 μL of FastPfu Polymerase, and roughly 10 ng of template DNA. The thermal cycling process entailed an initial denaturation at 95°C for 3 min, followed by 27 cycles of 30 s at 95°C for denaturation, 30 s at 55°C for annealing, and 45 s at 72°C for elongation, culminating in a final extension at 72°C for 10 min. PCR products were pooled in equal density ratios, purified using the AxyPrep DNA Gel Extraction Kit (Axygen Biosciences, Union City, CA), and quantified with a NanoDrop 8000. The purified amplicons were then pooled in an equimolar manner and subjected to paired-end sequencing (2 × 300) on an Illumina platform by Majorbio Bio Pharm Technology Co., Ltd. (Shanghai, China). Operational taxonomic units (OTUs) were clustered at a 97% sequence similarity threshold using UPARSE (version 11; http://drive5.com/uparse/uparse/), and taxonomic analysis of representative OTU sequences was performed with a 97% similarity threshold using the RDP classifier Bayesian algorithm.^1^

### 2.7 Statistical analysis

The conformity of residuals to normality and variance homogeneity was verified through visual inspection to confirm adherence to the assumptions of ANOVA (OriginLab version 9.75). A one-way Analysis of Variance (ANOVA) was conducted to assess the impact of treatment types (grazed vs. restored) on various parameters, including TBI parameters (*k* and *S*), aboveground biomass, soil properties, SOC fractions, and the diversity of AMF. Alpha diversity of the AMF community, represented by Chao1, Shannon, and Simpson indices, was calculated using QIIME (version 1.9.1) and R (version 2.15.3). Person correlation analysis was employed to explore the relationships between TBI parameters, aboveground biomass, soil properties, SOC fractions, and AMF diversity. For visual representation, all figures were created using Datagraph (Version 5.2, Visual Data Tools).

## 3 Results

### 3.1 Aboveground biomass

**Figure 2:**
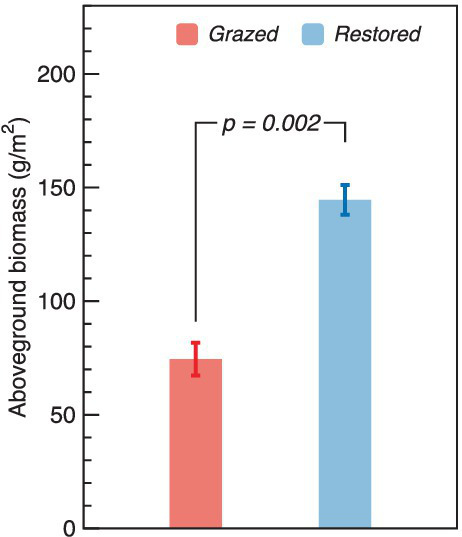
Comparison of aboveground biomass between grazed and restored sites. The data presented are means ± SE (*n* = 3). Differences between the two treatment groups were evaluated using Tukey’s test, with significance indicated by the corresponding *p*-value.

The restoration efforts in alpine wetlands have markedly enhanced aboveground biomass. The biomass in restored sites reached 144.56 g/m^2^, significantly surpassing the 74.52 g/m^2^ observed in sites subjected to livestock grazing. This indicates that restoration led to an aboveground biomass nearly double (1.94 times higher) that of the grazed sites (Figure 2).

### 3.2 Soil properties

The soil C and N contents of restored site were 1.33 and 1.49 times higher than that of grazed site, respectively (Table 1). Restoration also significantly influenced other soil properties, including soil pH, EC, and the contents of NH_4_^+^ and WSOC. Specifically, the NH_4_^+^ and WSOC contents in the restored site were 4.95 and 1.51 times higher than in the grazed site, respectively. Additionally, both EC and pH experienced a notable decrease in the restored areas, with reductions of 23.74 and 5.90%, respectively, relative to the grazed sites.

**Table 1:** Comparison of soil properties under grazed and restored sites.

Measurement	Grazed	Restored	*p* value
Total C (%)	6.18 ± 0.04	8.21 ± 0.66	**<0.05**
Total N (%)	0.39 ± 0.02	0.58 ± 0.06	**<0.05**
NH_4_^+^ (mg/kg)	4.32 ± 0.85	21.38 ± 4.10	**<0.05**
NO_3_^−^ (mg/kg)	1.71 ± 0.24	1.63 ± 0.30	0.83
WSOC (mg/kg)	1049.73 ± 92.49	1584.40 ± 151.62	**<0.05**
Soil moisture (%)	38.00 ± 3.51	46.50 ± 0.31	0.07
EC (μS/cm)	53.33 ± 1.45	40.67 ± 1.33	**<0.01**
pH	5.34 ± 0.11	5.03 ± 0.02	**<0.05**

WSOC is water soluble organic carbon, NH4+ is ammonium, NO3− is nitrate, EC is electrical conductivity. The table presents the mean ± SE (*n* = 3) for each parameter, significant *p* values are shown in bold letters based on the one-way ANOVA.

### 3.3 AMF diversity

**Figure 3:**
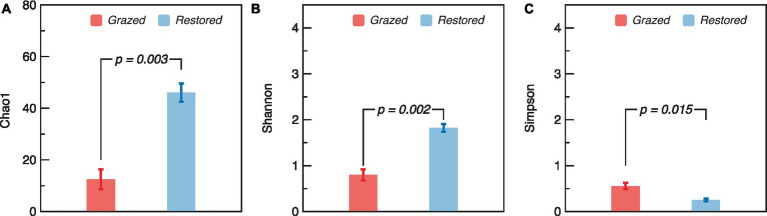
α-diversity of soil arbuscular mycorrhizal fungi (AMF) in grazed and restored sites. The data presented are means ± SE (*n* = 3). Differences between the two treatment groups were evaluated using Tukey’s test, with significance indicated by the corresponding *p*-value. Note: **(A)** Chao1 index, **(B)** Shannon Index, and **(C)** Simpson index.

**Figure 4:**
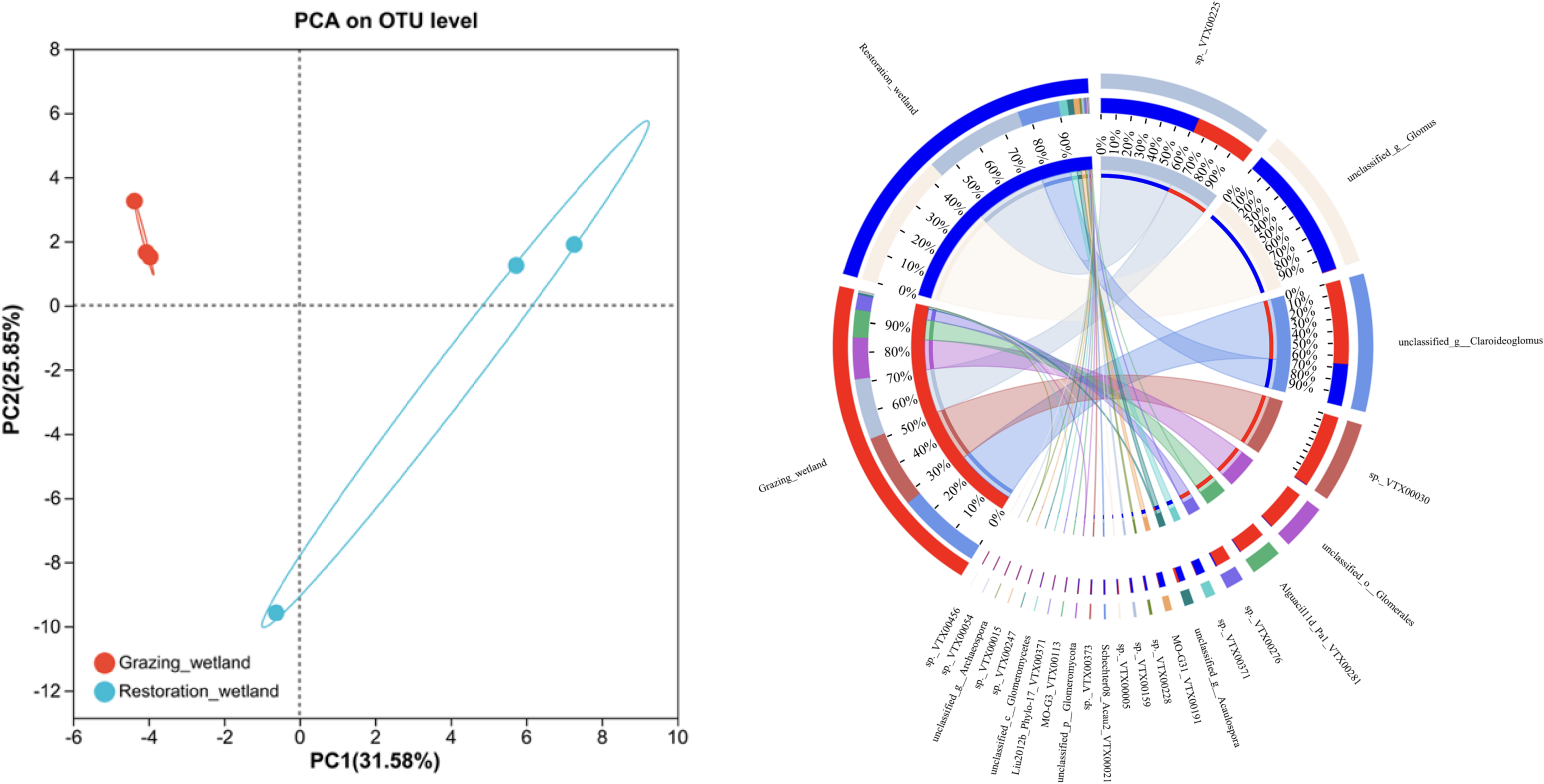
β-diversity of the soil arbuscular mycorrhizal fungi (AMF) under grazed and restored sites. The left panel shows the variations in the AMF community found in the grazed and restored sites, using non-metric multidimensional scaling (NMDS). The right panel displays the relative abundance of AMF at the genus level in the soils of the grazed and restored sites.

The richness and diversity of the soil AMF community displayed notable differences between grazed and restored sites. Specifically, the restored sites exhibited significantly higher values in the Chao1 and Shannon indices compared to the grazed sites (Figures 3A,B). In contrast, the Simpson index in the grazed sites, was significantly higher than that of in the restored sites (Figure 3C). The soil AMF community exhibited a clear distinction between the grazed and restored sites, as evidenced by the Non-metric Multidimensional Scaling (NMDS) results (Figure 4). In the restored sites, a dominant composition was observed with sp._VTX00225 (43%), unclassified_g_Glomus (35%), and unclassified_g_Claroideoglomus (12%) collectively making up approximately 90% of the AMF community. On the other hand, the grazed sites presented a different community structure, with unclassified_g_Claroideoglomus (34%), sp._VTX00030 (22%), sp._VTX00225 (20%), unclassified_o_Glomerales (12%), and Alguaci11d_Pal_VTX00281a (10%) together accounting for over 90% of the AMF composition. Notably, this composition in the grazed sites does not include the species unclassified_g_Glomus, which is a significant component in the restored sites. This variation highlights the influence of land management practices on the diversity and distribution of soil AMF species.

### 3.4 TBI parameters

**Figure 5:**
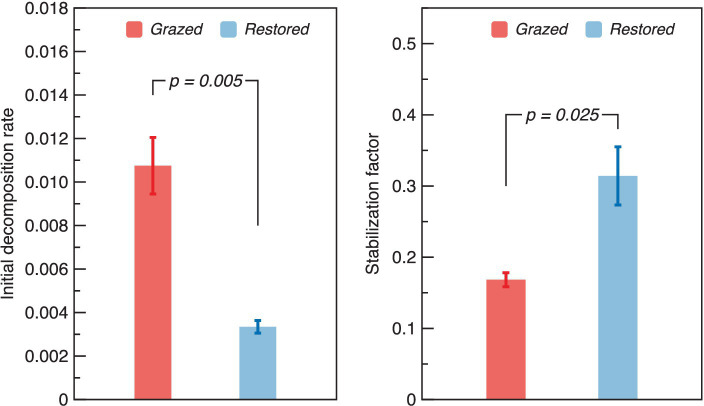
Variation of Tea bag index parameters (initial decomposition rate – *k* and stabilization factor - *S*) under grazed and restored sites. The data presented are means ± SE (*n* = 3). Differences between the two treatment groups were evaluated using Tukey’s test, with significance indicated by the corresponding *p*-value.

A substantial decrease in the *k* was detected after the restoration, which marking a reduction of 0.42 times compared to the grazed site (Figure 5). Conversely, the *S* in the restored site significantly increased, being 1.73 times higher than that observed in the grazed site.

### 3.5 POC and MAOC

**Figure 6:**
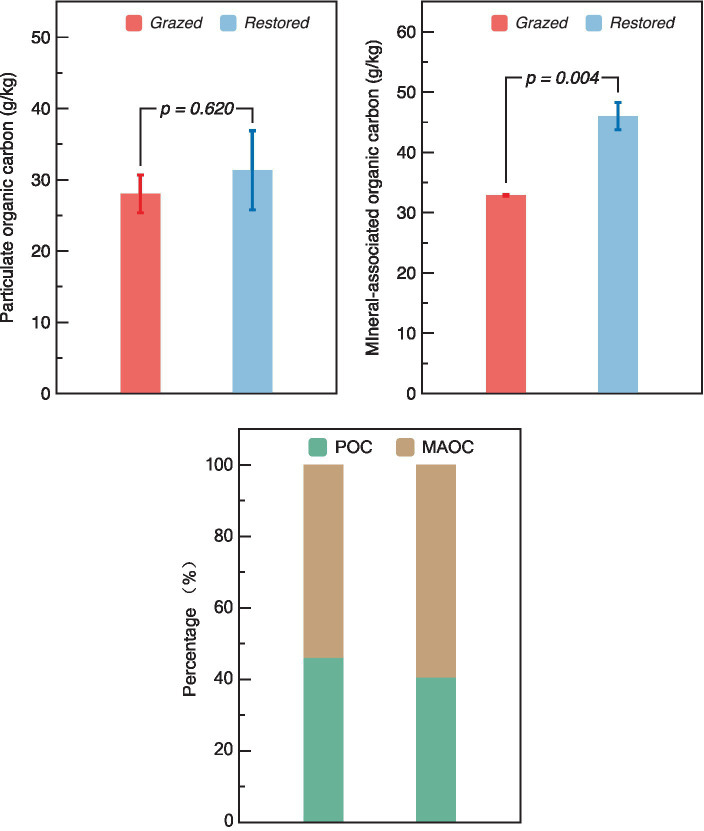
Comparison of particulate organic carbon (POC), mineral-associated organic carbon (MAOC) content and both fractions percentage in grazed and restored sites. The data presented are means ± SE (*n* = 3). The figure presents both the absolute content and the relative percentage distribution of POC and MAOC under the two treatment scenarios. Differences between the two treatment groups were evaluated using Tukey’s test, with significance indicated by the corresponding *p*-value.

Restoration efforts did not significantly alter the levels of POC between grazed and restored sites. However, there was a marked increase in the MAOC in the restored sites, where it reached 46.02 g/kg, demonstrating a 1.40-fold increase compared to the grazed sites. In terms of the proportion of the SOC fractions, the grazed site exhibited an equal distribution between POC and MAOC (Figure 6). In contrast, the restored site showed a higher percentage of MAOC relative to POC.

### 3.6 Factors controlling AMF Community and SOC fractions

**Figure 7:**
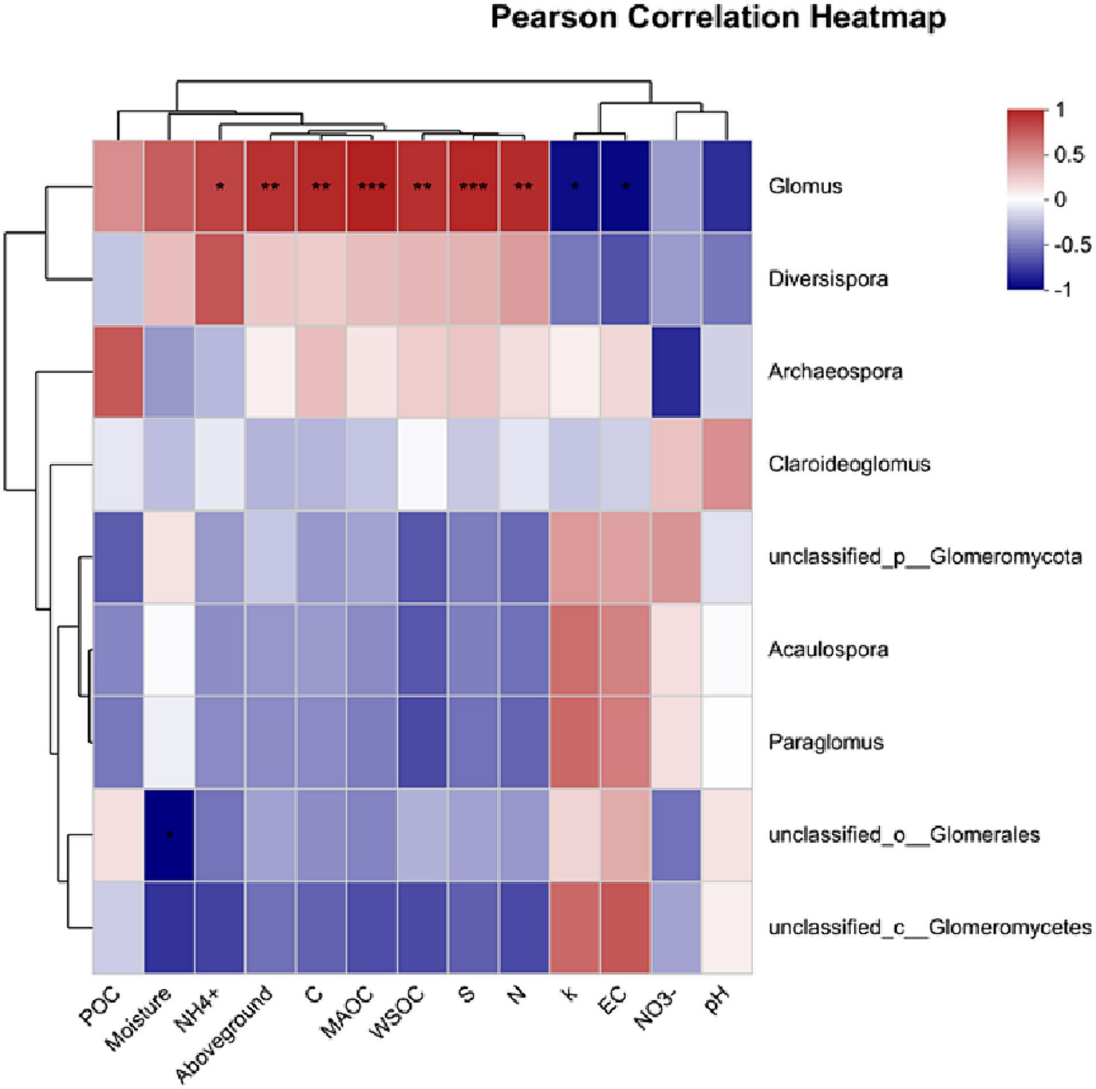
Factors controlling AMF community analyzed via Pearson correlation. POC, particulate organic carbon; WSOC, water soluble organic carbon; MAOC, mineral-associated organic carbon; S, stabilization factor; *k*-initial decomposition rate.

**Figure 8:**
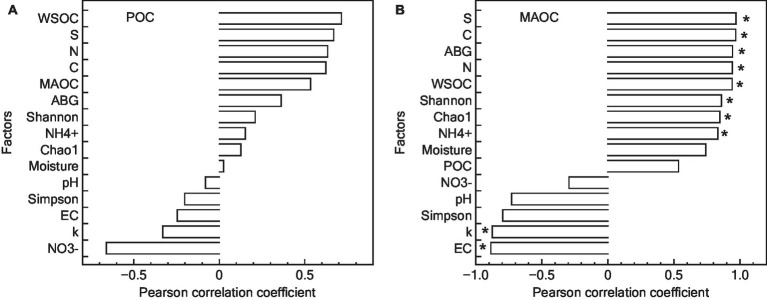
Pearson correlation analysis of factors controlling POC **(A)** and MAOC **(B)** content. POC, particulate organic carbon; ABG, aboveground biomass; WSOC, water soluble organic carbon; MAOC, mineral-associated organic carbon; S, stabilization factor; k-initial decomposition rate. *represented that the *p*-value for POC or MAOC in relation to other factors was statistically significant, according to the Pearson correlation analysis.

The genus Glomus was positively correlated with several variables including NH_4_^+^, aboveground biomass, total C and N content, MAOC, WSOC content, and *S* (Figure 7). Additionally, POC content did not show a significant relationship with examined factors (*p* > 0.05). In contrast, MAOC exhibited a significant positive correlation with various parameters including *S*, total C and N content, aboveground biomass, WSOC content, Shannon index, Chao1 index, and NH_4_^+^. Furthermore, MAOC was found to have a significant negative correlation with the *k* (Figure 8).

## 4 Discussion

### 4.1 Influence of restoration on plant biomass and soil properties

Alpine wetland restoration from grazing significantly increased aboveground biomass and influenced soil properties (including content of NH_4_^+^ and WSOC, EC, and pH). This finding supports our first hypothesis and is corroborated by multiple studies indicating that restoration activities contribute to improved soil quality and nutrient availability (Burden et al., 2019; Xu et al., 2019; Yang et al., 2019; Sea et al., 2022; Lovelock et al., 2023; Liu J. et al., 2023), thereby fostering greater plant growth and productivity as observed in the increased aboveground biomass at restored sites (Figure 2). Contrary to our expectations, however, NO_3_^−^ concentrations and soil moisture did not significantly differ between grazed and restored sites. While our hypothesis anticipated that restoration would lead to changes in these parameters, reflecting an overall improvement in soil health, the observed consistency suggests that some anticipated effects of restoration on nutrient cycling and water retention might be more nuanced in alpine ecosystems. The lack of significant change in NO_3_^−^ concentrations and soil moisture could be due to the intricate balance of nutrient cycling and water dynamics in these ecosystems, potentially moderated by external factors such as climatic conditions and inherent soil properties, which might not be directly influenced by short-term restoration efforts (Pärn et al., 2018; Pan et al., 2021). This insight highlights the complexity of ecological restoration impacts, emphasizing the need for a deeper understanding of how such interventions interact with the unique environmental contexts of alpine wetlands.

### 4.2 Impact of restoration on the diversity of the AMF community

The restored sites exhibited significantly higher values in the Chao1 and Shannon indices, which are measures of species richness and diversity, respectively. This finding is consistent with our first hypothesis, which predicted that restoration activities would positively impact the diversity of AMF in alpine wetland ecosystems. Such an increase in biodiversity is critical for maintaining a balanced and functional ecosystem, as diverse AMF communities are known to improve nutrient cycling and increased resilience to environmental stressors (George and Ray, 2023). To further explore the AMF community, our analysis indicated that restoration practices significantly increased the richness of the AMF community (Figure 4). This suggests that the restoration process contributes to the quantitative increase in AMF species. The enriched AMF community could lead to improved symbiotic relationships with plant roots, thus facilitating better nutrient uptake, enhancing plant growth, and contributing to the overall stability of the restored wetland ecosystem (Mai et al., 2023; Yang et al., 2023). This enrichment of the AMF community aligns with the ecological understanding that restoration interventions can create more favorable soil conditions, promoting a diverse and thriving microbial community (Van Geel et al., 2021). Furthermore, our results also found that the genus Glomus was positively correlated with several variables including NH_4_^+^ content, aboveground biomass, C content, MAOC, WSOC content, *S* and N content (Figure 7). This suggests that the presence and diversity of AMF (i.e., genus Glomus) is closely linked to improved soil properties, higher biomass production, and better nutrient cycling.

### 4.3 Effect of restoration on SOC fractions

Restoration significantly increased the MAOC content, while its influence on POC was less pronounced. This observation partly aligns with our last hypothesis. This inconsistent result could be attributed to the nature of the organic matter and the soil processes involved. POC is more decomposable organic matter, may not accumulate in the same manner, possibly due to faster decomposition rate (Witzgall et al., 2021; Feng et al., 2022). In wetland ecosystems, moisture is a crucial factor regulating the decomposition of POC, as it can alter certain key microenvironments in the soil. However, the results of this study found that short-term wetland restoration did not change the water condition, which may be a key reason for the unchanged POC content. Furthermore, POC content did not exhibit a strong correlation with litter turnover, soil properties, or AMF activity. These results indicated that the POC turnover could be influenced by factors not measured in this study, such as microsite variability, the presence of recalcitrant compounds within the POC fraction, or temporal shifts in microbial community composition and function (Condron et al., 2010; Bhattacharyya et al., 2022). Therefore, the observed lack of correlation underscores the need for a multifaceted approach to studying C stabilization, one that considers both the biochemical and physical facets of soil C dynamics. Future research should aim to untangle the intricate relationships between POC and these broader ecological processes, potentially leveraging advanced analytical techniques to capture the subtle yet critical ways in which POC contributes to the overall stability of soil C in restored alpine wetland ecosystems.

MAOC is typically associated with finer, mineral-bound organic particles and is more stable than POC (Georgiou et al., 2022). The restoration process may enhance the microbial activity and soil aggregation, which facilitates the incorporation of SOC into stable mineral complexes, thereby increasing MAOC (Liu et al., 2021). The results of this study showed that MAOC positively related with a range of factors including *S*, C content, N content, aboveground biomass, WSOC content, Shannon index, Chao1 index, and content of NH_4_^+^. Additionally, a significant negative correlation was observed between MAOC and *k*. These findings suggest that MAOC is more intricately linked to the restored ecosystem’s C dynamics. The positive correlations indicate that enhanced soil quality, increased AMF diversity, and higher biomass are conducive to the stabilization and retention of C in the form of MAOC (Liu et al., 2022; Su et al., 2023). The negative correlation with *k* further implies that slower decomposition rates in restored sites favor the long-term stabilization of SOC, emphasizing the role of MAOC as a critical component in soil C sequestration. Thus, our study not only highlights the importance of focusing on MAOC as a key indicator of C stabilization, but also suggests that AMF could play an important role in enhancing C stabilization within the context of alpine wetland restoration.

## 5 Conclusion

This study illustrates the significant impact of ecological restoration on the Zoige Alpine Wetlands, demonstrating improved soil properties, increased plant biomass, and enhanced soil C storage, particularly through the increase in MAOC. A notable finding is the crucial role of AMF diversity in supporting these positive changes. Our results highlight that restoration not only bolsters ecological balance but also promotes C stabilization, with AMF diversity playing a key role in this process. The use of the TBI provided new insights into SOC dynamics, showcasing a shift towards more effective C sequestration in restored areas. Consequently, this research underlines the value of restoration in mitigating climate change and enhancing ecosystem resilience, advocating for continued ecological restoration efforts as a strategy for environmental conservation and climate change mitigation.

## Data availability statement

The datasets presented in this study can be found in online repositories. The names of the repository/repositories and accession number(s) can be found in the article/Supplementary material.

## Author contributions

HT: Conceptualization, Data curation, Formal analysis, Funding acquisition, Investigation, Methodology, Validation, Visualization, Writing – original draft, Writing – review & editing. QL: Conceptualization, Data curation, Methodology, Validation, Writing – review & editing. QB: Data curation, Funding acquisition, Investigation, Writing – review & editing. BT: Investigation, Writing – review & editing. KL: Data curation, Investigation, Writing – review & editing. YD: Investigation, Methodology, Writing – review & editing. XL: Methodology, Writing – review & editing. QZ: Methodology, Writing – review & editing. SL: Investigation, Methodology, Resources, Writing – review & editing. XS: Methodology, Resources, Writing – review & editing. WL: Methodology, Writing – review & editing. LD: Conceptualization, Funding acquisition, Visualization, Writing – original draft, Writing – review & editing.

## Footnotes

^1^ http://rdp.cme.msu.edu/
