# Supplementary material for: Interplay of soil characteristics and arbuscular mycorrhizal fungi diversity in alpine wetland restoration and carbon stabilization
Source: Front Microbiol. 2024 Apr 10;15:1376418. doi: 10.3389/fmicb.2024.1376418 (PMC11039953; doi:10.3389/fmicb.2024.1376418)
Supplement: Supplementary file 1 [file Table_1.docx]

Table S1 Pearson correlation among Tea bag index parameters, soil properties, organic carbon fractions, and the diversity of arbuscular mycorrhizal fungi. Note: k-initial decomposition rate, S-stabilization factor, C- total carbon content, N-nitrogen content, WSOC-water soluble organic carbon, POC-particulate organic carbon, MAOC-mineral-associated organic carbon. Red color represents significant Pearson correlation values, and blue color indicates significant p values.

| Index | *k* | *S* | ABG | N | C | NH_4_^+^ | NO_3_^-^ | WSOC | Moisture | EC | pH | POC | MAOC | Chao1 | Shannon | Simpson |
| --- | --- | --- | --- | --- | --- | --- | --- | --- | --- | --- | --- | --- | --- | --- | --- | --- |
| *k* | 1 | **<0.05** | **<0.05** | **<0.05** | 0.08 | **<0.05** | 0.66 | **<0.05** | 0.27 | **<0.01** | 0.11 | 0.52 | **<0.05** | **<0.01** | **<0.01** | **<0.01** |
| *S* | -0.835 | 1 | **<0.05** | **<0.01** | **<0.01** | **<0.05** | 0.33 | **<0.01** | 0.18 | **<0.05** | 0.15 | 0.15 | **<0.01** | 0.07 | 0.07 | 0.10 |
| ABG | -0.905 | 0.868 | 1 | 0.05 | **<0.05** | 0.06 | 0.69 | **<0.05** | 0.15 | **<0.05** | **<0.05** | 0.48 | **<0.01** | **<0.05** | **<0.01** | **<0.01** |
| N | -0.834 | 0.985 | 0.807 | 1 | **<0.01** | **<0.05** | 0.35 | **<0.01** | 0.17 | **<0.05** | 0.21 | 0.18 | **<0.01** | 0.06 | 0.08 | 0.14 |
| C | -0.763 | 0.986 | 0.850 | 0.960 | 1 | 0.09 | 0.40 | **<0.01** | 0.14 | 0.07 | 0.19 | 0.13 | **<0.01** | 0.11 | 0.11 | 0.16 |
| NH_4_^+^ | -0.857 | 0.813 | 0.785 | 0.856 | 0.751 | 1 | 0.65 | 0.07 | 0.09 | **<0.01** | 0.10 | 0.77 | **<0.05** | **<0.01** | **<0.05** | 0.08 |
| NO_3_^-^ | 0.229 | -0.487 | -0.208 | -0.469 | -0.428 | -0.239 | 1 | 0.35 | 0.64 | 0.78 | 0.52 | 0.15 | 0.57 | 0.99 | 0.82 | 0.65 |
| WSOC | -0.851 | 0.982 | 0.817 | 0.984 | 0.958 | 0.782 | -0.468 | 1 | 0.24 | **<0.05** | 0.26 | 0.11 | **<0.01** | 0.07 | 0.08 | 0.12 |
| Moisture | -0.541 | 0.630 | 0.664 | 0.642 | 0.679 | 0.741 | 0.247 | 0.565 | 1 | 0.12 | 0.31 | 0.96 | 0.09 | 0.09 | 0.22 | 0.39 |
| EC | 0.960 | -0.842 | -0.859 | -0.873 | -0.781 | -0.950 | 0.151 | -0.850 | -0.704 | 1 | 0.14 | 0.64 | **<0.05** | **<0.01** | **<0.01** | **<0.05** |
| pH | 0.721 | -0.664 | -0.858 | -0.593 | -0.621 | -0.734 | 0.331 | -0.548 | -0.506 | 0.681 | 1 | 0.88 | 0.10 | 0.15 | 0.06 | **<0.05** |
| POC | -0.331 | 0.670 | 0.362 | 0.634 | 0.623 | 0.152 | -0.661 | 0.714 | 0.025 | -0.246 | -0.081 | 1 | 0.27 | 0.81 | 0.69 | 0.70 |
| MAOC | -0.876 | 0.971 | 0.944 | 0.942 | 0.968 | 0.833 | -0.294 | 0.940 | 0.742 | -0.885 | -0.728 | 0.535 | 1 | **<0.05** | **<0.05** | 0.06 |
| Chao1 | -0.939 | 0.770 | 0.849 | 0.799 | 0.716 | 0.924 | 0.008 | 0.775 | 0.750 | -0.986 | -0.663 | 0.126 | 0.848 | 1 | **<0.01** | **<0.05** |
| Shannon | -0.977 | 0.778 | 0.944 | 0.754 | 0.718 | 0.828 | -0.122 | 0.767 | 0.584 | -0.929 | -0.801 | 0.210 | 0.861 | 0.932 | 1 | **<0.01** |
| Simpson | 0.939 | -0.726 | -0.925 | -0.680 | -0.652 | -0.760 | 0.238 | -0.699 | -0.436 | 0.847 | 0.865 | -0.202 | -0.796 | -0.837 | -0.974 | 1 |
